# Relationship between early life asthma and chronic airway disease in adult life – in search for disease trajectories over the life span- the RELATE study based on the Kongsberg cohort

**DOI:** 10.1186/s12890-023-02661-8

**Published:** 2023-09-28

**Authors:** Osman Savran, Klaus Bønnelykke, Charlotte Suppli Ulrik

**Affiliations:** 1 Respiratory Research Unit, Department of Respiratory Medicine, Copenhagen University Hospital Hvidovre, Hvidovre, Denmark; 2 Institute of Clinical Medicine, University of Copenhagen, Copenhagen, Denmark; 3 Copenhagen Prospective Studies On Asthma in Childhood (COPSAC), Copenhagen University Hospital Gentofte, Gentofte, Denmark

**Keywords:** Early life, Childhood, Asthma, Chronic airway diseases, Prognosis

## Abstract

**Background:**

Chronic airway disease in adults may have its origin in early life. The purpose of this study is to investigate the long-term prognosis of severe childhood asthma in search for an association between asthma in early life and obstructive lung disease in adulthood.

**Methods:**

This study is based on the Kongsberg cohort, which includes approximately 5000 children with severe asthma with a 4-month stay at the asthma care facility in Kongsberg, Norway during the years 1950 to 1979. An on average 60-year observational study based on a follow-up examination will be performed including questionnaires, blood samples, and tests of lung function and bronchial responsiveness. Blood samples will be stored in a biobank. In addition, we will conduct further analyses of the cohort based on nationwide register data, including socio-economic parameters and mortality.

**Discussion:**

Chronic airway disease is associated with substantial burden for both the individual patient and society. Our knowledge of early life origins of chronic airway disease later in life has been increasing in recent decades but is still limited. By exploring early life risk factors for chronic airway disease in adulthood, we may gain insights paving the way for future reduction in the burden of chronic airway diseases.

## Background

Chronic airway diseases include diseases affecting airways and lung parenchyma with the most common being chronic obstructive pulmonary disease (COPD) and asthma [1]. COPD is globally the third leading cause of death, and is expected in the next decades to become the leading cause of death [2]. For decades, COPD has been regarded as a primarily smoking-related disease in adults with exposure to noxious particles and/or gases leading abnormalities of the airways and/or alveoli that cause persistent, often progressive, airflow obstruction [3]. Asthma has, on the other hand, been seen for decades as an atopic disease with onset early in life and characterized by variability in airway obstruction [4]. As opposed to COPD, mortality from asthma is low and most of the patients can live their life without asthma-related limitations [5]. In addition, previous studies have shown that the likelihood of remission from asthma is higher in children compared to adults [6]. A relationship between COPD and early life insults to lung function has previously received limited attention [7]. Observations from several studies in recent years have suggested that at least part of the determinants of chronic airway diseases in adulthood have its origin early in life [8–11]. It is, therefore, of outmost importance to identify the impact of potential insults in early life on lung health later in life. In line with this, childhood asthma, like other early life insults, has been proposed as a risk factor for adult COPD [12, 13].

Recent research have questioned the assumption that chronic airway disease follows a trajectory of excess lung function decline and suggests that low lung function in early life may also subsequently lead to chronic airflow limitation development later in life, not least in individuals with low lung function in early adulthood [9, 10, 14, 15]. In keeping with this, several long-term follow-up studies have investigated the association between childhood asthma characteristics and the presence of chronic airflow limitation later in life [12, 16–19]. A study including 317 children with a history of wheezing followed up to age 42, compared the cases with age-matched controls and found that almost three out of four individuals with frequent symptoms at seven years of age had recurrent symptoms of asthma, suggesting that the natural history of asthma depends on frequency and severity of symptoms during childhood [16, 17].

The East Boston Study investigated children aged five to nine followed for 13 years to assess lung function development in children with asthma and found that asthma was associated with an earlier onset of lung function decline in adulthood [20]. In addition, the Melbourne Asthma Study followed 458 children from the age of seven until the age of fifty [13]. The study cohort included five groups of children: controls, mild wheezy bronchitis, wheezy bronchitis, asthma, and severe asthma. At age fifty, follow-up revealed that the asthma and severe asthma groups had persistent airflow limitation [13].

The Childhood Asthma Management Program followed lung function in 1041 children with persistent asthma compared to lung function of children without asthma [19]. In a follow-up comprising almost two-thirds of the original cohort with at least one measurement of lung function between the age of 23 to 30, they found that lower level of lung function in childhood and being male were significant determinants of lower level of lung function compared to healthy age-matched controls [21]. Furthermore, a cross-sectional study of children with respiratory symptoms originally examined in 1964 were followed-up in 2001 including 177 individuals (63%) from the original cohort [22]. Analyses adjusted for smoking status, height, sex, and age revealed that adults with a history of childhood asthma had lower level of lung function and a significant excess lung function decline compared to controls.

In a recent prospective cohort study, Bui et al. (Tasmanian Longitudinal Health Study) followed 3609 participants from age 7 to 53 years and found that early-onset asthma that persisted into adulthood was associated with lower FEV1 and higher risk of COPD development in adulthood compared to late-onset asthma [23].

The prospective Tucson Children’s Respiratory Study followed 1246 healthy children from infancy to the age of 22 years [24]. Almost one-fifth of the children reported asthma symptoms at age 6 and almost twice as many reported asthma symptoms at some time point during follow-up in childhood [24].

Childhood asthma, as well as lung function impairment and COPD in adulthood, is highly influenced by genetic factors [25, 26], and it is likely that genetic susceptibility modifies the development of adult respiratory disease in children with asthma. Particularly genes related to lung function development, and the response to airway toxicants, are regarded as important.

The purpose of this study is to investigate the long-term prognosis of children with severe asthma in search for a link between childhood asthma and chronic airway diseases, that is asthma and COPD, in adulthood.

## Methods

### Aims

- Describe disease characteristics, including comorbidities, exacerbation history, lung function and airway responsiveness among elderly adults with a history of severe childhood asthma.
- Investigate long-term outcome of severe childhood asthma with regard to remission in late adulthood.
- Assess socioeconomic status factors among elderly adults with a history of childhood asthma compared to age-matched individuals with no previous diagnosis of obstructive airways disease.
- Determine factors associated with airflow limitation in elderly adults with a history of childhood asthma.
- Assess all-cause and disease-specific mortality in individuals with a history of severe childhood asthma.
- Examine genetic variations (SNPs) that may increase the risk of developing asthma and COPD during adult life.

### Study design

#### The Kongsberg cohort population

The Kongsberg cohort comprises approximately 5000 children with asthma that were referred to the Kongsberg asthma care facility, Norway, for a 4-month stay between 1950 and 1979 by physicians, primarily from the former Queen Louise’s Children’s Hospital in Copenhagen, Denmark. The Danish Red Cross managed the asthma care facility and organized the stays in Kongsberg. There are index cards and registration lists for all groups of children with a stay at Kongsberg, and by that the individuals comprising the Kongsberg cohort.

The available data from the children’s index cards and registration lists will be linked to each individual’s unique civil personal registration (CPR) number, a ten-digit number provided permanent residents in Denmark, in three steps. Firstly, we will digitalize data by entering lists with individuals’ data, that is name and date of birth, obtained from the index cards and registration lists for each group of children that was sent to Kongsberg, Norway. Secondly, from the unique civil personal registration database (in Danish called the CPR register), all CPR numbers of all individuals with the same name and date of birth of individuals appearing on index cards and registration list for the Kongsberg cohort will be extracted. Finally, the Danish Health Data Authority will match the CPR numbers extracted from the CPR register individual names and birth dates of the Kongsberg cohort. The Danish Health Data Authority will afterwards deliver CPR number of every individual of the Kongsberg cohort currently alive and residing in Denmark.

The eligible individuals with a previous stay at the Kongsberg asthma facility in childhood will be contacted by the research group for a follow-up examination program to assess current health status. In addition, the research group will apply for approval of access to data from national registries to investigate current socioeconomic status and mortality of individuals in the Kongsberg cohort.

#### Setting

This study comprises a follow-up examination program for individuals, who attended the Kongsberg asthma care facility in childhood due to severe asthma, as described in the ‘Examination program at study visit’, together with data obtained from nationwide registries, as described under ‘Registries’. The examination program will take place at The Respiratory Research Unit at Copenhagen University Hospital Hvidovre.

#### Registries

All hospital contacts, including primary and secondary diagnoses for each hospital contact, in Denmark are reported according to the International Classification of Diseases, 8^th^ revision (ICD-8) from 1977 to 1993 and from 1994 the 10^th^ revision (ICD-10) to the Danish National Patient Registry (DNPR), a part of Statistics Denmark. The DNPR has registered these data since 1977. The DNPR holds data on demographics, comorbidities, diagnoses, dates of admission and discharge from hospitals, and data for the registry data analyses will be provided from Statistics Denmark.

Data on filled prescriptions for each individual will be obtained from the Danish National Prescription Registry. The Danish National Prescription Registry comprises information on all prescriptions filled at national pharmacies since 1995. The available data includes information on date of dispensing, number of doses, strength, and the Anatomical Therapeutic Chemical (ATC) code. Statistics Denmark will also deliver data from the Danish National Prescription Registry for the analyses.

From Statistics Denmark, we will also obtain data from registries based on income, occupation (IND and LON) and education (UDDA). The data includes salaries, pensions (early retirement, which is granted to persons with a significant and permanently reduced ability to work, or voluntary early retirement), and levels of education. Registries on income and education comprise information on Danish residents since 1980.

Data on mortality of the Kongsberg cohort will be delivered from the Danish registry on cause of death (DAR), a part of the Danish Health Data Authority, which collects information from death certificates on all deaths in Denmark since 1970. The DAR holds data on causes of death, date of death, and age at time of death.

### Examination program at study visit

The following examinations and procedures will be carried out at the day of study visit. All procedures will be explained and demonstrated in detail to the participant prior to data being obtained:

1. Questionnaires
  - ACT (Asthma Control Test) [27, 28].
  - ACQ-7 (Asthma Control Questionnaire) [29].
  - CAT (COPD Assessment Test) [30].
  - CCQ (Clinical COPD Questionnaire) [31].
  - MRC (Medical Research Council) dyspnea scale [32].
  - Information will also be obtained for participants with regard to age, occupational history, marital status, lifestyle (including smoking habits, physical activity, alcohol use), comorbidities, including asthma, COPD, diabetes, cardio-vascular disease, osteoporosis, eczema, rhinitis, sleep-apnea, cataract, depression, and anxiety, and height and weight measurements, exacerbations in the past 12 months, prescribed medication for obstructive lung disease at the time of follow-up, unscheduled contacts with their GP for their chronic airways disease within the past 12 months, visits to the emergency room or hospital admission for chronic airway disease within the past 12 months.
2. Blood samples: C-Reactive Protein (CRP), alpha1-antitrypsin, white cell count and differential, red cell count, hemoglobin, vitamin D, and serum total IgE. Approximately 200 ml of blood will be collected from each participant for analysis. Blood samples will either be analyzed when collected or stored as a research biobank throughout the project period and destroyed according to Danish legislation.
3. Fractional exhaled nitric oxide: fractional exhaled nitric oxide (F_E_NO) will be measured with EcoMedics CLD 88sp analyzer and DENOX 88 at a controlled flow rate of 50 ml/s according to American Thoracic Society/European Respiratory Society (ATS/ERS) guidelines, and the mean value of two measurements in parts per billion (ppb) will be recorded [33].
4. Skin prick test: the skin prick test (SPT) will be performed according to the European Academy of Allergy and Clinical Immunology [34, 35]. The test will be carried out with 9 different aeroallergens: birch, grass, mugwort, dog, cat, dust mites (Dermatophagoides pteronyssinus and D. farinae), and moulds (Alternaria alternata and Cladosporium herbarum). The SPT will be positive if the wheal diameter is ≥ 3 mm after 15 min to at least one of the aeroallergens, and atopy is defined as a positive SPT [36].
5. Spirometry: measurement of forced expiratory volume in the first second (FEV_1_) from a maximum inspiration, forced vital capacity (FVC) and calculation of the FEV_1_/FVC ratio (Vitalograph 6800 pneumotrac) according to ERS/ATS guidelines [37, 38]. The participant will have three consecutive spirometry measurements. The reproducibility requirements must be met by the two best measurements. The best FEV_1_ and FVC values should be no more than 5% or 150 mL apart. Predicted FEV_1_ will be calculated based on height, age and sex [39].
6. Bronchodilator reversibility test: the bronchodilator reversibility test will be a measurement of FEV_1_ before and after administration of 0.4 mg of salbutamol. A positive reversibility test will be defined as an improvement of 12% and ≥ 200 ml in FEV_1_ from the pre-bronchodilator value.
7. Body Plethysmography: measurement of vital capacity (VC), and static lung volumes, including residual volume (RV), and total lung capacity (TLC) using a whole-body plethysmograph (Vitalograph 6800 pneumotrac) and according to ERS/ATS guidelines [37, 38].
8. Diffusion capacity (DL_CO_): diffusion capacity will be measured by the single-breath carbon monoxide gas transfer method together with the diffusion constant KCO. The participant inhales a gas mixture containing 0.3% carbon monoxide (CO), 9.3% helium, 21% oxygen and nitrogen. All results will be presented as percent predicted [40].
9. Bronchial challenge test: the mannitol bronchial provocation test will be performed using the commercial mannitol kit Osmohale© Pharmaxis (Pharmaxis Ltd, Australia) [41]. The kit provided for the bronchial challenge test includes filled capsules of mannitol in increasing doses and a dry powder inhaler. The challenge is carried out as previously described [42]. An evaluation of the safety of mannitol as a bronchial challenge test has previously been published [43]. If needed, rescue bronchodilator (0.2 mg salbutamol) will be provided at the end of the challenge test for aided recovery.
10. A genome-wide scan will be performed on EDTA blood samples in order to investigate genetic variants (SNPs) expected to influence airway development with a focus on SNPs related to asthma or COPD. This will be done using a modified version of the Infinium Global Screening Array. The method applied is, therefore, not considered extensive genetic mapping according to the genome guidelines. The analyses will be performed at the national State Serum Institute (SSI) department for neonatal screening.

### Definitions

#### Remission of asthma

Childhood asthma remission is defined as no use of asthma medications and no reported asthma symptoms within the previous 12 months [44].

#### Severe asthma

Severe asthma is defined as asthma that is treated with high-dose inhaled corticosteroids plus a second controller (or systemic corticosteroids for more than 50% of the last 12 months) or which remains ‘uncontrolled’ despite this therapy [45].

### Study endpoints

The primary endpoint will be to determine prevalence of childhood asthma remission in adult participants with previous severe childhood asthma in a follow-up examination.

The secondary endpoint is to quantify prevalence of airflow limitation and characteristics, including socioeconomical status in included population of adults with a history of childhood asthma.

The last endpoint is to determine mortality and genetic susceptibility associated with obstructive lung disease development in included study cohort.

### Sample size calculation

For sample size calculation, the study cohort including eligible elderly adults with previous severe childhood asthma was compared to a known value of asthma remission prevalence in a previous study sample [46]. The previous study based on the Melbourne Asthma Study found 10% asthma remission in those with a history of severe childhood asthma. In order to calculate sample size with the absolute error of 5% and including type 1 error of 5%, the following formula is used [47]:

$$\mathrm{Sample\;size}={(\left(1.96\right)}^{2}*0.1*(1-0.1))/{\left(0.05\right)}^{2}=139$$

A sample size consisting of at least 139 subjects would be sufficient to provide required statistical power for the primary endpoint.

### Statistical analyses

Asthma remission prevalence, factors associated with airflow limitation, and disease characteristics including comorbidities, exacerbation history, lung function, and airway responsiveness among elderly adults with a history of severe childhood asthma will be reported as mean values ± one standard deviation (SD) and clinical variables will be compared using independent sample t-test for continuous variables and chi-square for binary variables.

The comparison between characteristics including socioeconomical status in the study cohort compared to age-matched individuals with no previous record of obstructive airways disease will be conducted using a logistic regression model and reported as odds ratio (OR) and 95% confidence intervals (CI). A *p*-value < 0.05 will be considered statistically significant.

The hazard ratio (HR) and 95% CI by Cox regression will be used to estimate the association between mortality and disease characteristics.

Genetic effect modification will be analyzed based on candidate variants as well as combined polygenic risk scores for asthma and COPD.

Data will be analyzed using the statistical program R Statistics 3.61 software (R Foundation for Statistical Computing, Vienna, Austria).

## Discussion

Both asthma and COPD include great risk of mortality world-wide and are associated with substantial burden for both the individual patient and society [48]. Studies from recent years suggest that chronic airway disease in adults often have its origin in childhood [10–12, 49]. Perhaps most important are the results presented by Tai et al. where children with severe asthma were at increased risk (OR 32, 95% CI 3–269) of adult chronic airflow obstruction. Notably, in those with severe childhood asthma, 15% had a remission of asthma and 44% had chronic airflow obstruction by age 50. In contrast, of those with no childhood asthma, merely 8% had chronic airflow obstruction in adulthood [12]. It is, therefore, imperative to identify the impact of potential insults in early life on lung health later in life. However, a relationship between childhood asthma and chronic airway diseases in adulthood is still not well-established and more research, particularly prospective studies looking into the natural history of childhood asthma, is necessary. Furthermore, very few studies have investigated whether adults with a history of childhood asthma have a higher disease burden, mortality, and lower socio-economic status compared to adults with no previous record of asthma.

The interest in the early origins of chronic airways disease is built upon studies that support the hypothesis that there is a ‘window of opportunity’ for promoting respiratory health by preventing incomplete lung growth in early years and thus reduce incidence of adult chronic airway disease [13, 24, 50]. However, the complete underlying mechanism of childhood asthma increasing risk of severe lung disease in later life has not been established. A few studies suggest an increased airway resistance persists in childhood severe asthma causing a predisposition for chronic airway diseases in adulthood [21, 51]. The current study is based on a very large cohort of children (the Kongsberg cohort) with severe asthma and more than 60 years of follow-up. Examining this particular cohort, therefore, provides a unique opportunity to follow the pattern of disease, including non-respiratory diseases, from early childhood to the end of life. Furthermore, the comprehensive examination program included in the project, in addition to a history of severe childhood asthma, could provide more detailed information on the course of life-threatening obstructive lung disease.

Some limitations and strengths in our study are worth highlighting. The data from the cohort that will be obtained from the DNPR mostly originates from patient care in hospitals. However, studies using registries with insufficient data collection may be ineffective in obtaining data [52]. It is therefore plausible that data on disease and severity in eligible individuals treated solely by general practitioners in primary care will not be included. Data from the Danish National Prescription Registry comprises information on all prescriptions filled at national pharmacies and data from this registry is not limited to either primary or secondary care.

There is also selection bias in our study as it was children with severe asthma, who were selected for a referral to the asthma care facility in Kongsberg, Norway. However, we include individuals from a universal health care system where health care data is available in nationwide registries, which would usually reduce selection bias that may otherwise occur due to selective inclusion from certain hospitals, based on specific income levels, or specified age groups. In addition, the risk of recall bias in our study is very low because the national registries record data daily [53].

Another clear limitation of the present study will be relying on a correct diagnosis of severe childhood asthma made many years ago, when methods and definitions differed from practice today. On the other hand, the current study provides detailed knowledge of the course of childhood asthma towards the end of life, and not least details on factors that may positively affect the course, which may be crucial for future opportunities such as implementation of prevention strategies aiming at reducing the very high burden of disease related to chronic airway diseases. The Kongsberg cohort constitutes a specific group of individuals, so the current study results might not be completely generalizable. The associations found could be impacted by differences in lifestyle such as tobacco smoke exposure and genetic predispositions in the cohort. In addition, some of the children from the Kongsberg cohort have passed away or moved abroad and are naturally not eligible for study participation. Some individuals in the Kongsberg cohort may live far away from the site from where examination takes place, and this may keep them from participating in our follow-up examination program. Other factors that may prevent participation are severity of disease and old age. These factors may further decrease the external validity of the study. Additionally, the Kongsberg cohort was diagnosed with asthma by a physician before attending the asthma care facility, and children who had symptoms, which could be managed by a stay at the asthma care facility in Kongsberg, were chosen to attend the asthma care facility. This may have overestimated the number of children with severe asthma.

In this study protocol paper, we have presented our upcoming studies focusing on describing the Kongsberg cohort, investigating the comparison between the Kongsberg cohort and the background population, and determining factors associated with development of chronic airway diseases in adulthood. We also plan further studies on genetic susceptibility to obstructive lung disease development in addition to assessment of mortality risk in the Kongsberg cohort. An investigation of characteristics through an extensive examination combined with data from registries will help identify patients in the clinic with poor health status and high morbidity due to lung disease, and in the future hopefully, lead to better understanding of the long-term prognosis of childhood asthma.

## Data Availability

The datasets used and/or analysed during the current study will be available from the corresponding author on reasonable request.

## Abbreviations

COPD: Chronic Obstructive Pulmonary Disease
CPR: Civil Personal Register number
ACT: Asthma Control Test
ACQ-7: Asthma Control Questionnaire
CAT: COPD Assessment Test
CCQ: Clinical COPD Questionnaire
MRC: Medical Research Council
GP: General Practitioner
CRP: C Reactive Protein
IgE: Immunoglobulin E
F_E_NO: Fractional Exhaled Nitrogen Oxide
ATS: American Thoracic Society
ERS: European Respiratory Society
Ppb: Parts per billion
FEV_1_: Forced expiratory volume in the first second
FVC: Forced vital capacity
VC: Vital capacity
RV: Residual volume
TLC: Total lung capacity
DLCO: Diffusion capacity
CO: Carbon monoxide
SNP: Single nucleotide polymorphism
SSI: State’s Serum Institute (Danish: “Statens Serum Institut”)
SD: Standard Deviation
OR: Odds ratio
CI: Confidence Interval
